# Trimethylamine modulates dauer formation, neurodegeneration, and lifespan through *tyra‐3/daf‐11* signaling in *Caenorhabditis elegans*

**DOI:** 10.1111/acel.13351

**Published:** 2021-04-05

**Authors:** Amit Khanna, Durai Sellegounder, Jitendra Kumar, Manish Chamoli, Miguel Vargas, Shankar J. Chinta, Anand Rane, Christopher Nelson, T. Harshani Peiris, Rachel Brem, Julie Andersen, Gordon Lithgow, Pankaj Kapahi

**Affiliations:** ^1^ Buck Institute for Research on Aging Novato CA USA; ^2^ Dovetail Genomics LLC Scotts Valley CA USA; ^3^ Touro University California Vallejo CA USA

**Keywords:** Aging, *C. elegans*, chemotaxis, dauer, dopaminergic neurons, Parkinson's disease, Trimethylamine (TMA), TYRA‐3

## Abstract

In the nematode *Caenorhabditis elegans*, signals derived from bacteria in the diet, the animal's major nutrient source, can modulate both behavior and healthspan. Here we describe a dual role for trimethylamine (TMA), a human gut flora metabolite, which acts as a nutrient signal and a neurotoxin. TMA and its associated metabolites are produced by the human gut microbiome and have been suggested to serve as risk biomarkers for diabetes and cardiovascular diseases. We demonstrate that the tyramine receptor TYRA‐3, a conserved G protein‐coupled receptor (GPCR), is required to sense TMA and mediate its responses. TMA activates guanylyl cyclase DAF‐11 signaling through TYRA‐3 in amphid neurons (ASK) and ciliated neurons (BAG) to mediate food‐sensing behavior. Bacterial mutants deficient in TMA production enhance dauer formation, extend lifespan, and are less preferred as a food source. Increased levels of TMA lead to neural damage in models of Parkinson's disease and shorten lifespan. Our results reveal conserved signaling pathways modulated by TMA in *C. elegans* that are likely to be relevant for its effects in mammalian systems.

## Graphical abstract

TMA alters development, aging and neurodegeneration in *C. elegans*. Observed results suggests that the bacterial metabolite TMA alters development, aging and neurodegeneration in *C. elegans* through DAF‐11 and DAF‐16 pathways.

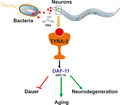

## 1 INTRODUCTION

The nematode *C. elegans* assesses environmental cues, including food availability, temperature, and population density to determine favorable conditions for reproduction (Golden & Riddle, 1982). *C. elegans* attains reproductive maturity under favorable conditions, but under adverse environments, worms enter dauer diapause, a long‐lived stress‐resistant stage (Fielenbach & Antebi, 2008). Both the nutritive quality and distinct chemical signals derived from bacterial food play a crucial role in developmental decisions (Gusarov et al., 2013; Khanna et al., 2016; Zecic et al., 2019). To detect microbial metabolites in the environment, *C. elegans* has evolved multiple mechanisms that allow discrimination between bacterial species that are appropriate food sources or pathogenic (Hu, 2007; J. Wang et al., 2013). This is in part achieved by a highly developed chemosensory system that perceives olfactory and gustatory cues emanating from potential food sources, such as bacteria, to determine optimal food choice (Fielenbach & Antebi, 2008). This complex assessment of local environmental conditions involves chemosensory inputs that feed into transforming growth factor‐beta (TGF‐β) and insulin‐like signaling (ILS) pathways. These ultimately converge to modulate the activity of key molecular factors such as forkhead transcription factor (DAF‐16/FOXO) (Avery & You, 2012). DAF‐16 activity is pivotal in determining the organism's response to either proceed with reproductive growth or to enter the dauer stage (Hu, 2007; Ogg et al., 1997). Although the molecular consequences of changes in food availability have been well studied, there is little understanding of specific bacterial signals that direct these choices in the host. *C. elegans* is known to respond to signals emanating from bacteria to detect food sources, and bacterial fatty acids can act as a signal that modulates recovery from the dauer stage (Kaul et al., 2014). A genetic screen for bacterial genes has identified several other signals including cAMP that in part direct these choices (Khanna et al., 2016; Urrutia et al., 2020).

In this study, we describe a role for trimethylamine (TMA) as a bacterial signal that both promotes food‐seeking behavior and activates nutrient signaling pathways. A gut microbiome‐derived metabolite, TMA, is produced from dietary phosphatidylcholine and is converted to trimethylamine N‐oxide (TMAO) in the body (Gipson et al., 2008; Z. Wang et al., 2011). Multiple studies have identified TMA or TMAO as a risk factor for type II diabetes and cardiovascular diseases in humans (Gipson et al., 2008; Z. Wang et al., 2011). Recent study reported increased gut‐derived TMAO in the cerebrospinal fluid of Alzheimer's disease (AD) patients with mild cognitive impairment emphasizing the role of this metabolite in connecting gut‐brain axis (Vogt et al., 2018). However, the mechanisms by which TMA and TMAO influence host molecular pathways and their physiological consequences remain poorly understood.

Here, we demonstrate that TMA acts as a nutrient signal in *C. elegans* sensed by the G protein‐coupled receptor (GPCR), TYRA‐3, and the guanylyl cyclase, DAF‐11, to modulate food perception, dauer formation, and lifespan. We additionally show that TMA causes dopaminergic neuronal loss in a TYRA‐3‐dependent manner. Given the conservation of molecular pathways modulating nutrient perception and signaling (Hu, 2007), *C. elegans* constitutes a powerful system for understanding how bacterial signals interact with their host, which is likely to be relevant for better understanding host‐microbiome interactions and their influence on healthspan in humans.

## 2 RESULTS

### 2.1 A conserved GPCR, *tyra‐3*, mediates TMA sensing in *C. elegans*

**FIGURE 1:**
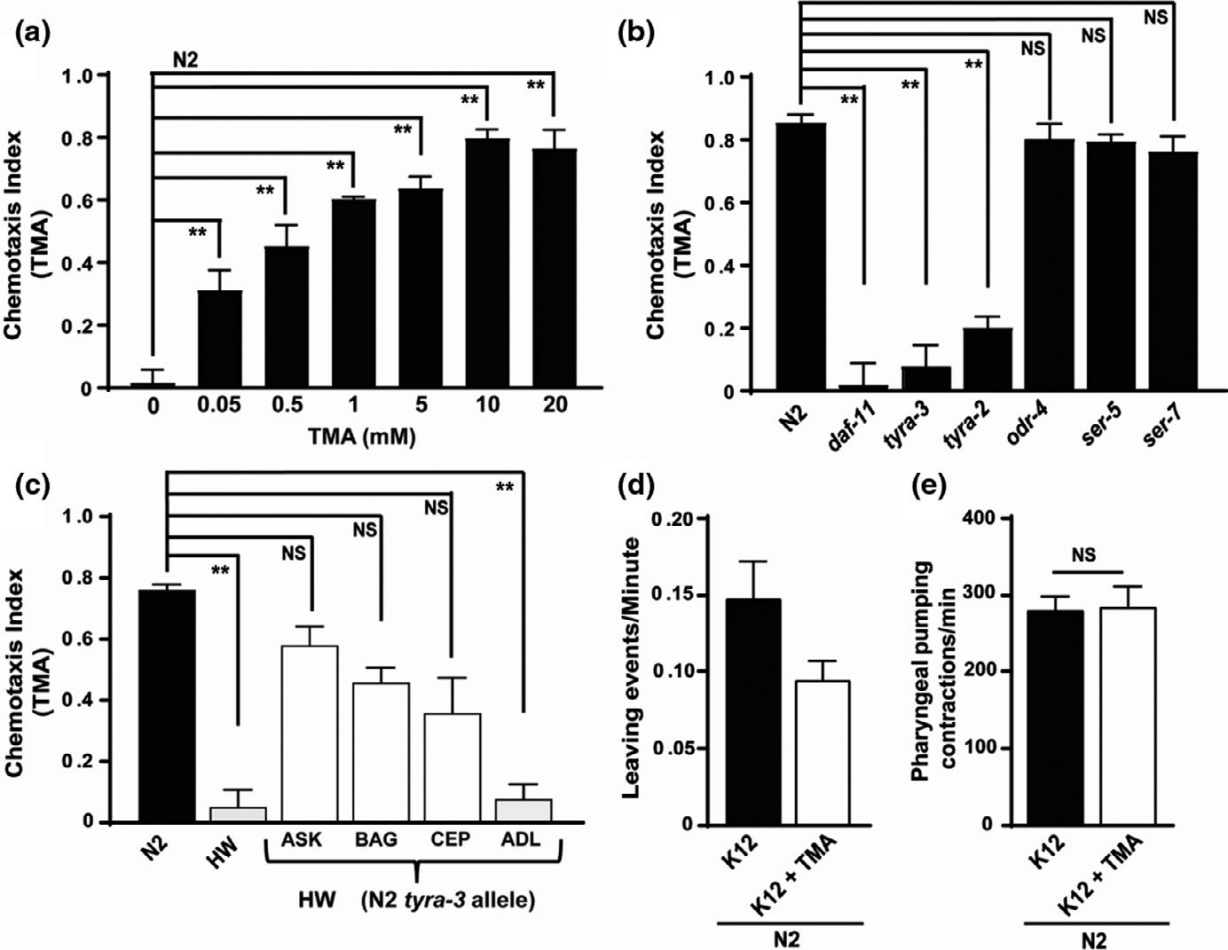
*C. elegans* require TYRA‐3, a GPCR, for sensing TMA. a. Synchronized day 1 age worms were tested for olfactory plasticity in the presence of TMA. Chemotaxis index (CI) for N2 worms in the presence of different concentrations of TMA. b. CI for N2, *daf*‐*11(m47)* mutant, and biogenic amine receptors mutants *odr‐4(n2144), ser‐7(tm1325), ser‐5(ok3087), daf‐11(m47), tyra‐3(ok325), and tyra‐2*(RNAi) in the presence of TMA (2 mM). c. CI for N2, Hawaiian (HW) and HW transgenic worms expressing the N2 *tyra*‐*3b* allele in ASK, BAG, or CEP neurons in the presence of TMA (2 mM). d. Lawn‐leaving events per minute for N2 worms were placed on K12 bacterial lawns in the presence and absence of TMA (2 mM). e. Pharyngeal pumping of N2 worms in presence of TMA (2 mM). Worms *n* ~ 300, *n* ≥ 6 assays, **p < 0.01; average ±std. dev (*n* = 3)

*C. elegans* possesses an intricate chemosensory system capable of distinguishing various chemical stimuli. *C. elegans’* response to food sources, toxins, or bacterial‐derived metabolites can be measured via chemotaxis assays (Bargmann, 2006; Bargmann et al., 1993; Margie et al., 2013). To evaluate whether TMA acts as a chemoattractant or a nutritional signal, we used a chemotaxis assay to measure *C. elegans* behavior in the presence of TMA. Wild‐type *C. elegans* (N2) were found in this assay to be attracted to TMA in the absence of bacteria in a dose‐dependent manner (Figure 1a; Table S1a).

*C. elegans* detect various chemical stimuli through specialized biogenic amine receptors (Troemel et al., 1995). These are typically GPCRs, and their signaling is conserved between *C. elegans* and vertebrates (Sengupta, 2007). To identify putative receptors involved in sensing TMA, we tested candidate mutants deficient in biogenic amine receptors using the chemotaxis assay. Observed results demonstrated that the tyramine receptor mutant *tyra‐3(ok325)* was unable to sense TMA. Similarly, RNAi‐mediated knockdown of *tyra‐2* reduced chemotaxis behavior toward TMA (Figure 1b). *C. elegans* *daf*‐*11(m47)* mutants containing a mutation in a transmembrane guanylyl cyclase also displayed defective chemosensory behavior (Figure 1b). This suggests that multiple receptors in *C. elegans* may sense TMA. In contrast, our candidate screen showed that loss of function in *odr*‐*4(n2144)* (odorant response abnormal protein 4) and the serotonin/octopamine receptor family genes *ser*‐*5(ok3087)* and *ser*‐*7(tm1325)* did not alter chemotaxis behavior toward TMA (Figure 1b). In order to rule out that changes in levels of *tyra*‐*3* accounted for lack of change in chemotaxis behavior in these mutants, we quantified *tyra*‐*3* expression via qRT‐PCR. Our results show that *tyra*‐*3* expression is unchanged in *ser*‐*5(ok3087)*, *ser*‐*7(tm1325)*, and *odr*‐*4(n2144)* mutants (Figure S1).

*C. elegans* *tyra*‐*3* is predicted to encode a GPCR for the invertebrate neurotransmitter tyramine (Wragg et al., 2007) and shares high conservation with the transmembrane domain of the human trace amine receptor (TAAR5) (WormBase). CB4856, a *C. elegans* strain isolated in Hawaii (HW), encodes a *tyra*‐*3* variant shown to be partially responsible for increased food leaving behavior (Bendesky et al., 2011). Since *C. elegans* tendency to leave or remain on a lawn of bacteria is linked to their capability to sense external stimuli, we performed chemotaxis assay on the HW worm strain. We found that HW worm was not attracted to TMA, further linking *tyra*‐*3* to the sensing of TMA (Figure 1c; Table S1). TYRA‐3 is expressed in the gonads, vulva, and several head neurons and in interneuron SDQ. To identify the functional loci involved in TYRA‐3‐mediated response to TMA, we used transgenic HW worms expressing N2‐derived *tyra*‐*3*b promoter (N2 *tyra*‐*3*b) in order to rescue variations in the non‐coding region of the HW strain (Bendesky et al., 2011). This sequence was driven by cell type‐specific promoters for BAG, CEP, ASK, or ADL neurons in biogenic amine receptor mutant worms. Transgenic expression of N2 *tyra*‐*3*b in ASK, BAG, and CEP neurons, but not ADL, partially rescued TMA attraction in HW worms (Figure 1c; Table S1). This confirms the necessity of a functional TYRA‐3 in ASK, BAG, and CEP neurons for positive chemotaxis behavior in response to TMA.

*C. elegans* display behavioral responses to bacterial food based on both the quality of food available and the animal's ability to detect the food. If food is easily available, the worm will spend more time on the bacterial lawn, a phenotype called dwelling. If the food is not of a preferred variety, worms avoid the bacterial colony and search for more preferable food, a phenotype called roaming (Shtonda & Avery, 2006). The food intake in *C. elegans* can be measured by counting the pharyngeal pumping rate. We used lawn‐leaving behavior and pharyngeal pumping to test the ability of TMA to alter both food‐seeking and food intake behaviors. Results demonstrate that addition of 2 mM TMA to bacterial food significantly reduced lawn‐leaving behavior (Figure 1d) without altering food intake (Figure 1e).

### 2.2 TMA requires TYRA‐3 and acts through DAF‐11 to regulate dauer formation in *C. elegans*

**FIGURE 2:**
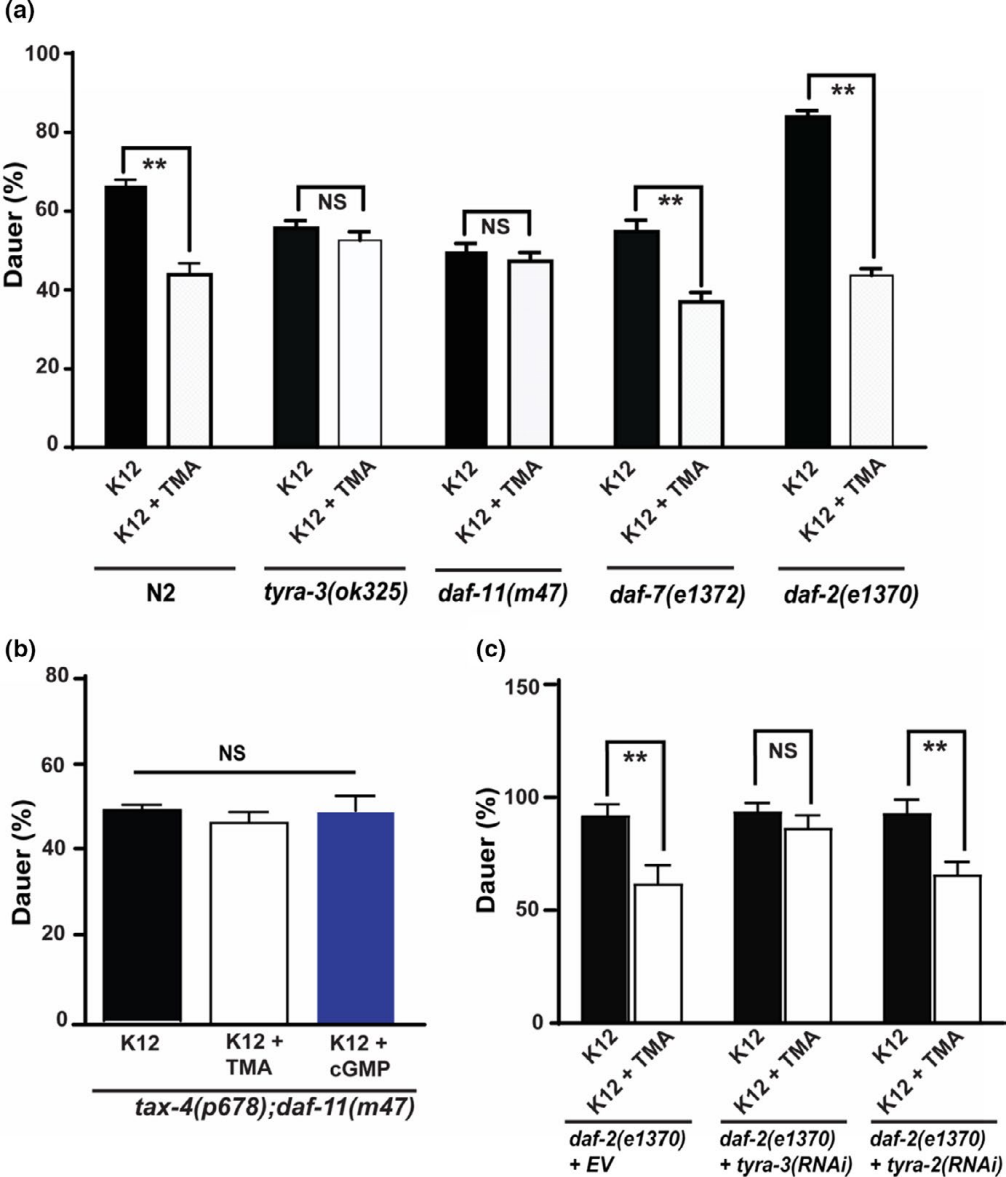
TMA modulates dauer formation in *C. elegans*. a. Age‐synchronized worms were tested for dauer formation on minimal media plates without glucose on K12 bacterium lawns at high temperature (as specified in materials and methods) in the presence and absence of TMA (2 mM). b. Under similar assay conditions, dauer formation was quantitated in the presence of cGMP (2 mM) in eggs from *daf*‐*11(m47)*, and *tax*‐*4*;*daf*‐*11* double mutant worms. c. Dauer assays were performed with synchronized *daf*‐*2(e1370)* mutant worms grown on bacteria expressing worm RNAi for *tyra*‐*3*, *tyra*‐*2*, and vector control. Eggs were exposed to K12 bacteria in the presence and absence of TMA, and dauers were scored. **p < 0.001; average ±std. dev (*n* = 3)

*C. elegans* proceed through four predetermined larval stages (L1–L4). However, under stressful conditions (e.g., low food or high temperature), L1 larvae arrest development and trigger a non‐feeding, diapause stage termed dauer larvae (L2d) (Cassada & Russell, 1975). Increased food availability or favorable changes in the environment initiate feeding and molting of L2d larvae to L4. Given the role of TMA as a food signal, we examined whether TMA could act as a nutrient signal to influence dauer formation. To avoid confounding effects of dietary amines (or lack thereof), we employed minimal media conditions (no glucose) and high temperature (Lee et al., 2009) to examine the effects of exogenous TMA (2 mM) on dauer formation. The addition of TMA to K12 bacteria lawns significantly reduced dauer formation in N2 (by ~23%) compared to control, whereas treatment of *tyra*‐*3(ok325)* mutant worms with TMA showed no significant decrease in dauer formation (Figure 2a; Table S2a). This supports the hypothesis that TMA requires *tyra*‐*3* to influence dauer signaling.

Current understanding of dauer formation and aging in *C. elegans* has revealed a complex interconnected network of signal transduction pathways consisting primarily of the guanylyl cyclase (*daf*‐*11*), TGF‐β (*daf*‐*7*), and insulin‐like signaling (ILS) (*daf*‐*2*) pathways (Hu, 2007). We interrogated the involvement of TMA in these key pathways using mutant *C. elegans* strains. Under minimal media conditions, addition of TMA (2 mM) to K12 bacterial lawns reduced dauer formation in N2 (by ~23%), *daf*‐*2(e1370)* (by ~40%), and *daf*‐*7(e1372)* (by ~17%) compared to control but had no effect on *daf*‐*11(m47)* mutants (Figure 2a; Table S2a). These results suggest that TMA functions through the DAF‐11 pathway to rescue *C. elegans* from entering the dauer larval stage.

In *C. elegans*, the functional loss of DAF‐11 signaling leads to a reduction in intracellular cGMP and enhanced dauer formation (Beckert et al., 2013), while addition of exogenous cGMP reduces dauer formation in *daf*‐*11(m47)* mutants (Birnby et al., 2000). DAF‐11 mutants are known to inhibit dauer arrest via activation of the cyclic nucleotide‐gated channel beta subunits TAX‐2 and TAX‐4 through increased cGMP synthesis (Hu, 2007). We therefore tested the role of TAX‐4 in this pathway by generating *tax*‐*4(p678)*;*daf*‐*11(m47)* double mutants. We found that the addition of TMA (2 mM) or the cGMP analog, 8‐bromo‐cGMP (2 mM), inhibited dauer in *daf*‐*11(m47)* single mutant but did not inhibit dauer formation in the *tax*‐*4(p678)*;*daf*‐*11(m47)* double mutant under minimal media conditions (Figure 2c; Table S2a), suggesting that TAX‐4 is required for TMA‐induced DAF‐11 signaling. Since TAX‐4 is known to display abnormal thermotaxis and chemotaxis behavior (Komatsu et al., 1996), we did not include this single mutant in the assay.

Addition of 8‐bromo‐cGMP was found to reverse the reduction in dauer formation observed in *tyra*‐*3(ok325)* mutants, suggesting that TYRA‐3 is upstream of the guanylyl cyclase pathway (Table S2a). TMA (2 mM) did not rescue dauer formation in *daf*‐*2(e1370)* worms with a *tyra*‐*3* knockdown compared to *daf*‐*2(e1370)* worms alone or *daf*‐*2(e1370)* worms with a *tyra*‐*2* knockdown (Figure 2d). Taken together, our results show that TMA is sensed by TYRA‐3 and modulates dauer formation through DAF‐11 signaling while also interacting with DAF‐2 pathway.

Guanylyl cyclase, TGF‐β, and insulin‐like signaling (ILS) pathways are known to independently modulate DAF‐16, a *C. elegans* forkhead (FOXO) homolog that influences both dauer formation and lifespan (Hahm et al., 2009; Hu, 2007). Upon mild heat shock, DAF‐16 translocate from the cytoplasm to the nucleus to modulate gene expression including those involved in dauer formation (Lee et al., 2009). To interrogate the potential role of TMA in the DAF‐16 pathway, we utilized a DAF‐16 reporter strain and explored its response to mild temperature increase in the presence of TMA. We observed that exposure to TMA significantly increased cytoplasmic retention of DAF‐16 in worms shifted from 15ºC to 21.5ºC in a dose‐dependent manner (Figure S2). Together, these results indicate that TYRA‐3 acts upstream of the dauer signaling pathway(s) to modulate both dauer formation and chemotaxis behavior in response to biogenic amines like TMA.

### 2.3 Bacteria‐specific DMSO reductase mutant modulates nutrient sensing and dauer formation in *C. elegans*

**FIGURE 3:**
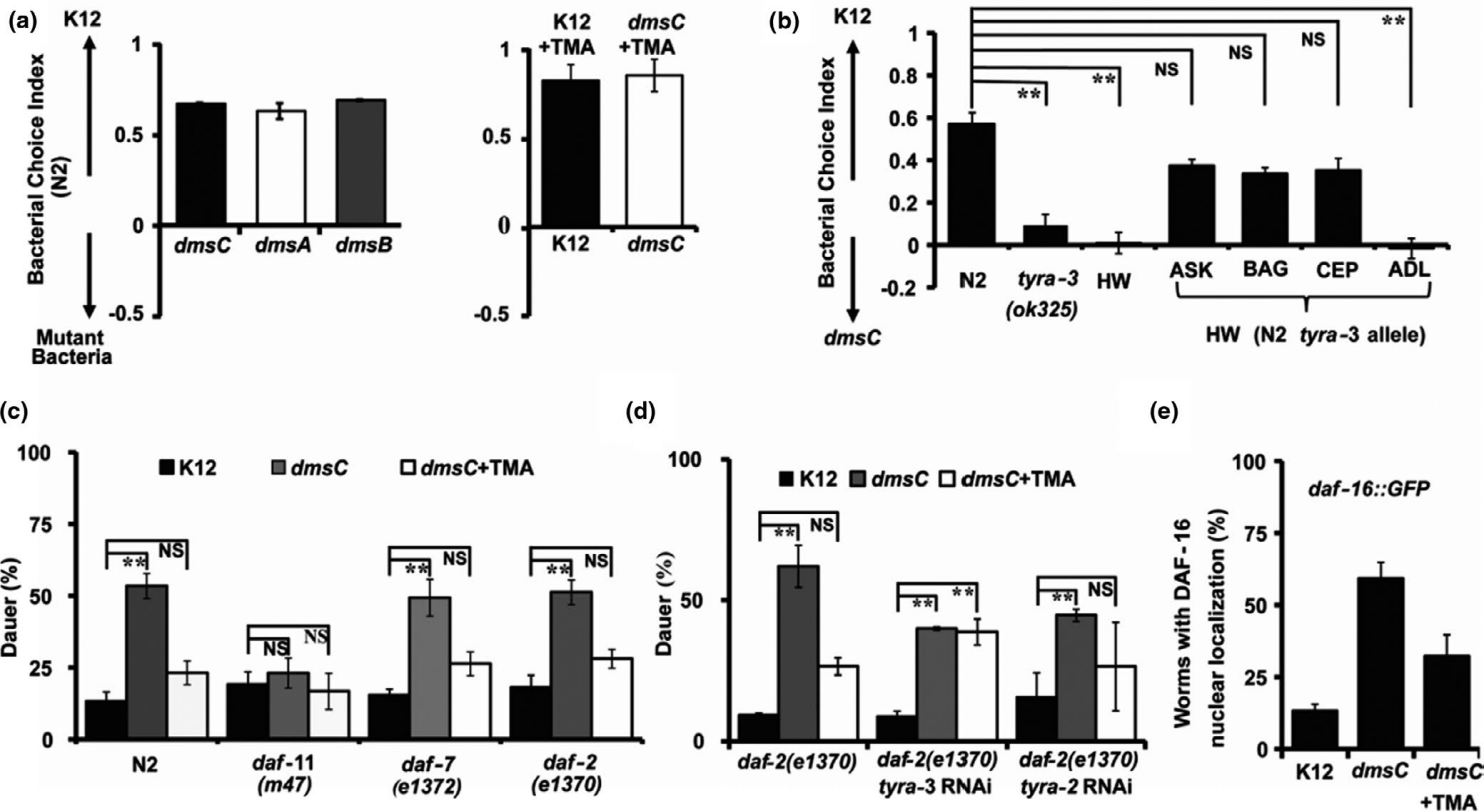
Bacteria‐specific DMSO reductase mutant modulates nutrient sensing and dauer formation in *C. elegans*. a. Bacterial choice index for N2 worms exposed to K12 and DMSO reductase (*dms*) mutants in the presence or absence of TMA (2 mM). b. Bacterial choice index for HW, *tyra*‐*3(ok325)* mutant, and HW transgenic worms expressing the N2 *tyra*‐*3b* allele in ASK, BAG, or CEP neurons in the presence of wild‐type (K12) bacteria as compared to *dmsC* mutant bacteria. Worms *n* ~ 300, *n* ≥ 6 assays. **p < 0.01; average ±std. dev (*n* = 3). c. Dauer assays were performed on minimal media plates without glucose with K12 bacterium lawns at 21.5°C in the presence and absence of TMA (2 mM). Synchronized eggs were used from N2 (wild‐type), *daf‐2(e1370), daf‐7(e1372), and daf‐11(m47)* mutant worms. d. Dauer assays were performed with eggs from *daf*‐*2(e1370)* mutants worms kept on bacteria expressing worm RNAi for *tyra*‐*3*, *tyra*‐*2*, and vector control. Eggs were exposed to K12 and *dmsC* mutant bacteria in the presence and absence of TMA and dauers scored. e. DAF‐16::GFP nuclear localization in worms fed K12 *vs* *dmsC* mutant bacteria in the presence or absence of TMA (2 mM). Worms *n* ~ 300, *n* ≥ 6 assays, **p < 0.01; average ±std. dev (*n* = 3)

Bacteria utilize dimethyl sulfoxide (DMSO) and TMA as oxidizing agents. The bacterial enzymes DMSO reductase (*dms*) and trimethylamine N‐oxide (TMAO) reductase are involved in catalyzing the reduction of various N‐oxides to amines, such as TMAO to TMA (Baba et al., 2006; Cammack & Weiner, 1990; Sambasivarao & Weiner, 1991). To examine whether bacteria with altered amine production are sufficient to influence nutrient‐sensing pathways in *C. elegans*, we screened single‐gene mutants (Figure S3a) of DMSO reductase (*dms*)/TMAO reductase and genes involved in choline and betaine metabolism (Table S2b). Using a bacterial choice assay (Glater et al., 2014), we found that N2 worms were more attracted to K12 (control) bacteria than to DMSO reductase mutant bacteria (*dmsA*, *dmsB*, *dmsC)* with or without TMA (Figure 3a; Table S2b). Consistent with their defect in TMA perception, *tyra*‐*3(ok325)* and HW animals showed no difference in attraction to these two different bacterial strains (Figure 3b). However, HW transgenic worms expressing N2 *tyra*‐*3b* allele in either ASK, BAG, or CEP neurons preferred K12 over *dmsC* mutant bacteria (Figure 3b). Neither *daf*‐*11* nor *tyra*‐*3* mutants displayed a significant preference for K12 bacteria over *dmsC* mutant bacteria as compared to N2 worms (Figure S3b, c). We observed a significant decline in lawn‐leaving events in N2 worms when *dmsC* bacterial lawns were supplemented with TMA (Figure S3d).

Next, we assessed the potential role of DMSO reductases in dauer formation in N2, *daf*‐*11(m47)*, *daf*‐*7(e1372)*, and *daf*‐*2(e1370)* mutant worms. Since the goal of this assay is to understand the role of bacterial enzymes and TMA, we performed dauer assay using minimal media conditions (no glucose) at 21.5ºC. We observed a significant increase in dauer formation in N2 worms fed with bacteria lacking DMSO/TMAO reductases. Interestingly, supplementing *dmsC* mutant lawns with TMA (2 mM) significantly decreased dauer formation compared to *dmsC* mutant controls (Figure 3c). We also observed a significant increase in dauer formation in *daf*‐*7(1372)* and *daf*‐*2(e1370)* mutant worms which was rescued by addition of TMA (2 mM). No significant change was observed in *daf*‐*11(m47)* mutant worms under these conditions, further supporting a role for DAF‐11 signaling in TMA‐dependent dauer formation (Figure 3c). The HW parent strain does not sense food and did not show dauer formation when fed with *dmsC* mutant bacteria. However, HW worms expressing the N2 *tyra*‐*3b* allele in ASK, BAG, or CEP neurons showed an increase in dauer formation when worms were fed with *dmsC* mutant bacteria (Table S2c). Addition of 2 mM TMA significantly rescued this dauer phenotype (Table S2c), suggesting that TMA act as nutrient signal in rescuing *C. elegans* from dauer development and that *tyr*‐*3* is required to sense TMA.

The addition of TMA (2 mM) or a cGMP analog (2 mM) to *dmsC* mutant bacterial lawns did not decrease dauer formation in the *tax*‐*4(p678)*;*daf*‐*11(m47)* double mutant under semi‐permissive conditions (Figure S3e). Similarly, we observed that TMA (2 mM) did not rescue dauer formation in *daf*‐*2(e1370)* worms with a *tyra*‐*3* knock down compared to *daf*‐*2(e1370)* worms or *daf*‐*2(e1370)* worms with a *tyra*‐*2* knockdown when fed on *dmsC* mutant bacteria (Figure 3d), confirming the role of *daf*‐*11* signaling and emphasizing the requirement of *tyra*‐*3* for sensing TMA. In addition, we observed increased DAF‐16 nuclear localization in worms fed with *dmsC* mutant bacteria which was rescued by addition of exogenous TMA (2 mM) (Figure 3e). No dauers were observed in the dauer defective, *daf*‐*16(mu86)*, worms fed with *dmsC* mutant bacteria, indicating that DAF‐16 is required to manifest the effects of *dmsC* mutant bacteria on dauer formation (Data Not shown). These results suggest that TMA triggers a neuroendocrine signal that modulates DAF‐16 activity to promote reproductive growth and inhibit dauer formation. Our results support the idea that biogenic amines including TMA play an important role in *C. elegans* nutrient‐sensing pathways by signaling the presence of bacteria and that TMA signaling is mediated in part through TYRA‐3 to modulate both dauer formation and chemotaxis behavior.

### 2.4 TYRA‐3 mediates TMA‐dependent dopaminergic neuronal damage

**FIGURE 4:**
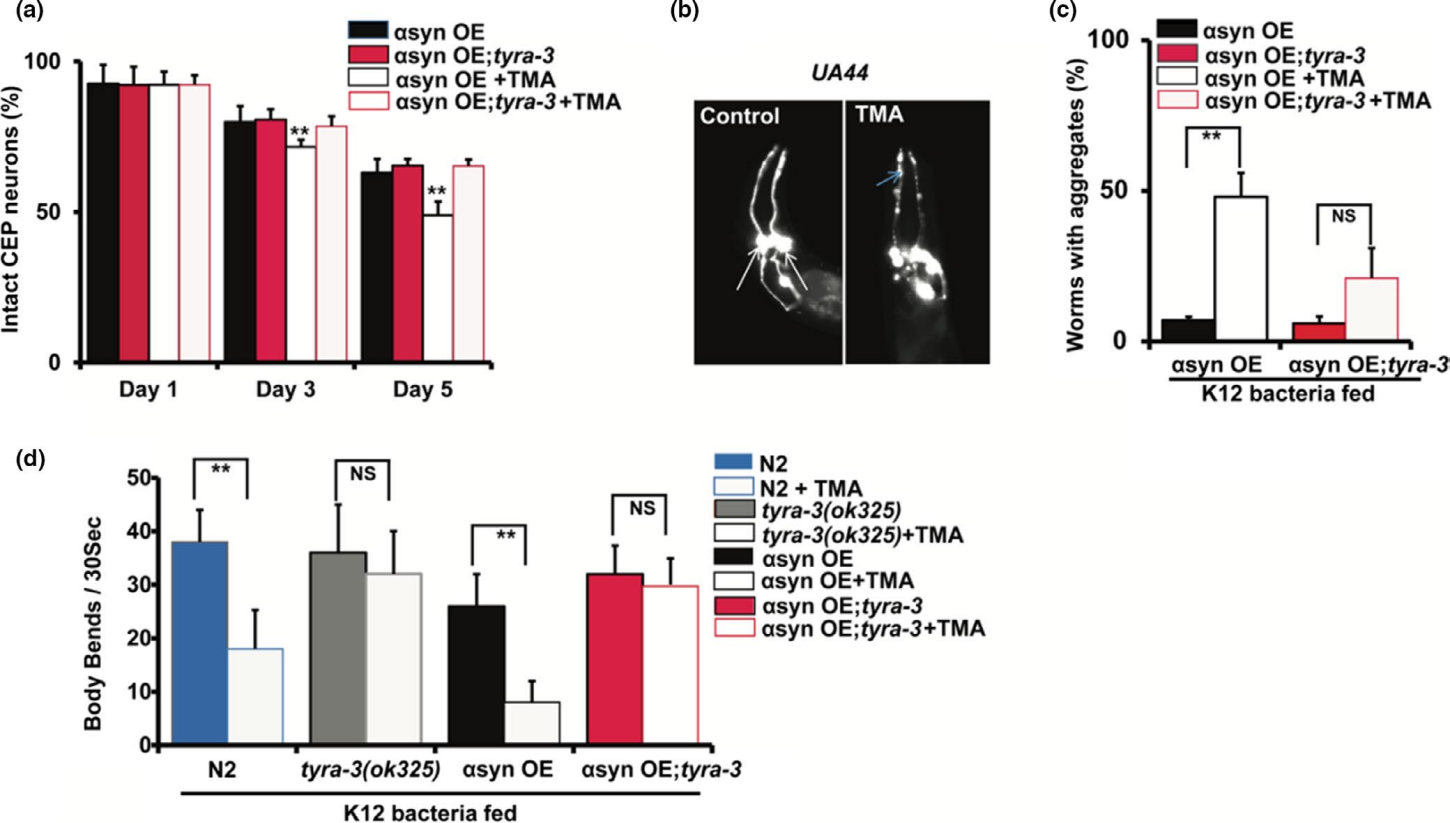
TYRA‐3 mediates TMA‐dependent dopaminergic neuronal damage. a. Histogram shows the percentage of intact CEP neurons/all worms scored on day 1, 3, and 5 in αsyn OE and αsyn OE;*tyra*‐*3* transgenic worms after exposure to TMA (2 mM). b. Representative images of αsyn OE expressing αsyn and GFP specifically in the four anterior DA neurons in Control (water) and TMA (2 mM). c. Histogram shows the number of aggregates in DA dendrites after exposure to TMA for 5 days in αsyn OE and αsyn OE;*tyra*‐*3*. d. Number of body bends are presented as a histogram for wild‐type worms (N2), *tyra*‐*3* (*ok325*), αsyn OE, and αsyn OE;*tyra*‐*3* transgenic in absence and presence of TMA (2 mM). Worms *n* ~ 120, *n* ≥ 6 assays. **p < 0.01; average ±std. dev (*n* = 3)

TYRA‐3 is expressed in several neurons including the CEP dopaminergic (DA) neurons (Bendesky et al., 2011). Our results in Figure 1 and Table S2c provide evidence that *tyra*‐*3* expression in CEP neurons is relevant for the TMA‐induced chemotaxis behavior and dauer formation. Therefore, we further wished to investigate whether TMA could affect dopaminergic (DA) neuronal health. For this purpose, we used the *C. elegans* strain UA44 [baInl1 (P*_dat_*_‐_ *_1_*::αsyn, P*_dat_*_‐_ *_1_*::GFP)] which overexpressed human α‐synuclein with GFP reporter in the four anterior DA neurons to test for effects in the presence of TMA (2 mM). The strain UA44 is represented as αsyn OE in this manuscript. We observed a significant (~16%) decline in intact DA neurons in day 3 and day 5 old αsyn OE worms upon exposure to TMA (Figure 4a). However, transgenic αsyn OE;*tyra*‐*3* (UA44 with *tyra*‐*3(ok325)* mutation) did not show a significant decline in intact DA neurons upon exposure to TMA (2 mM) (Figure 4a). Addition of TMA (2 mM) significantly increased aggregation in dendrites by day 3 in αsyn OE worms compared to controls (Figure 4b). However, strain αsyn OE;*tyra*‐*3* did not show a significant change in aggregation compared to controls when treated with TMA (2 mM) (Figure 4c).

*C. elegans* placed in liquid demonstrates specific locomotor behavior dubbed “body bends” or “thrashing.” Thrashing behavior or body bends are regulated by motor neurons and therefore can be used to assess motor neuron function (Matthies et al., 2006). We counted the number of body bends per 30 seconds in the presence and absence of TMA. Here, we observed a significant decline in body bends in WT and αsyn OE worms treated with 2 mM TMA compared to the non‐treated (Figure 4d). Conversely, *tyra*‐*3* mutant worms treated with 2 mM TMA did not decrease body bend rate, implying that TYRA‐3 is relevant for the TMA‐induced loss of neurons (Figure 4d). Our results demonstrate that TMA causes an increase in alpha‐synuclein aggregation and a loss in DA neurons through its receptor TYRA‐3. Further, knockdown of *tyra*‐*3* was sufficient to negate the effects of TMA‐induced alpha‐synuclein aggregation and loss of neuronal function (Figure 4d).

### 2.5 TMA shortens *C. elegans* lifespan in a *tyra*‐3‐ and *daf*‐*11*‐dependent manner

**FIGURE 5:**
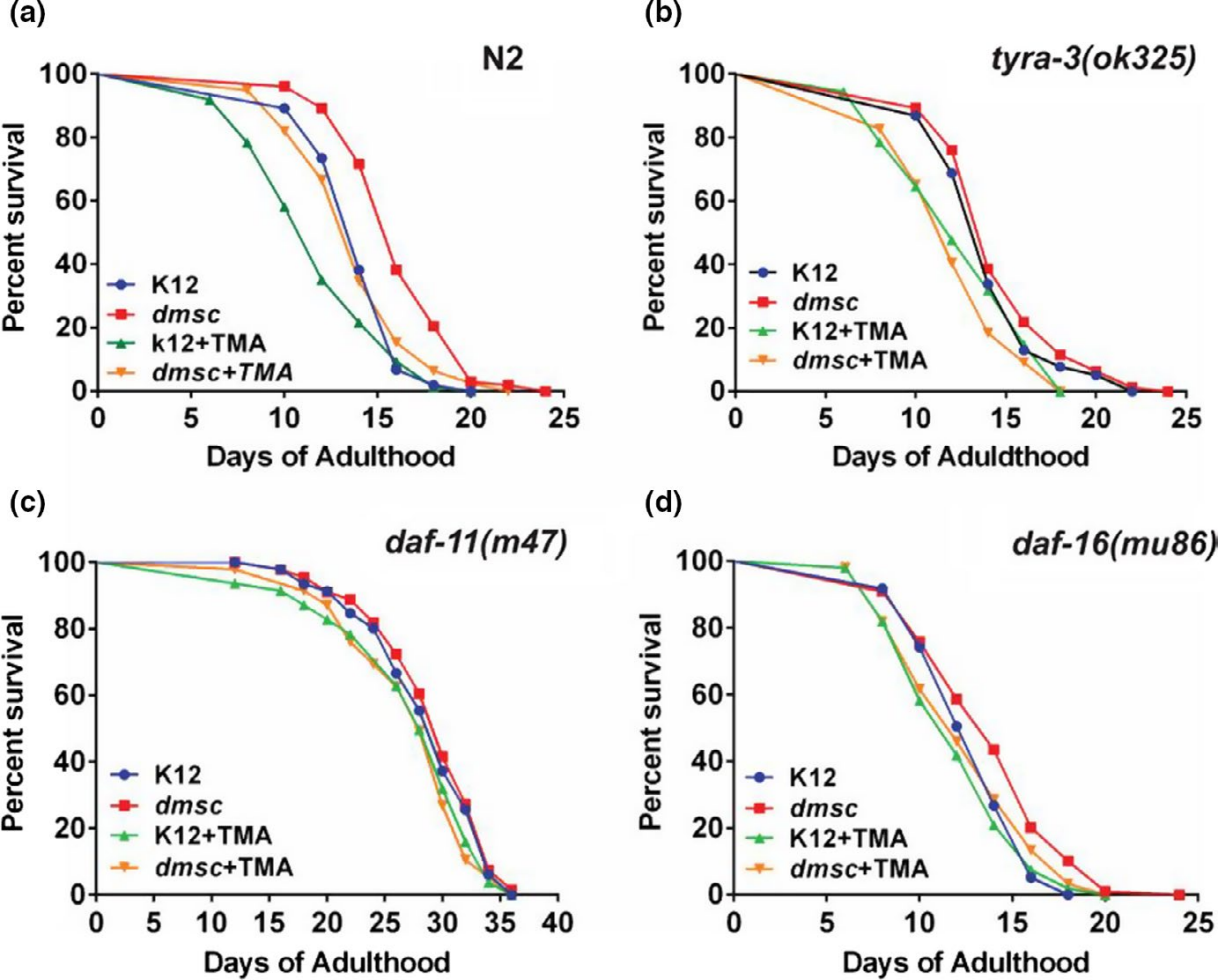
TMA modulates lifespan. A‐D. Kaplan–Meier survival curves of synchronously aged hermaphrodite N2, *tyra*‐*3(ok325)*, *daf*‐*11(m47)*, and *daf*‐*16(mu86)*, mutant worms fed K12 or *dmsC* mutant bacteria in the presence or absence of TMA (2 mM). a. N2 K12 vs *dmsC* (*p* < 0.001); K12 vs K12+TMA (*p* < 0.001). b. *tyra*‐*3(ok325)* K12 vs *dmsC* (*p* = 0.2197); K12 vs K12+TMA (*p* < 0.001; *dmsC* vs *dmsC* +TMA (*p* < 0.001) (*p* < 0.001). c. *daf*‐*11(m47)* K12 vs *dmsC* (*p* = 0.6314). d. *daf*‐*16(mu86)* K12 vs *dmsC* (*p* = 0.0027); *dmsC* vs *dmsC*+TMA (*p* = 0.0009). Graphs represents average of two independent experiments. Worms (*n*) ~80–120, **p < 0.001; (*n* = 2)

As nutrient and sensory cues play an important role in modulating longevity (Bargmann, 2006; Bendesky et al., 2011; Libina et al., 2003), we examined the effects of TMA on worm lifespan. We observed a significant increase in the mean lifespan (15.61%) of N2 worms fed *dmsC* mutant bacteria compared to K12 bacterial‐fed worms (Figure 5a). In addition, we found that mean lifespan was significantly reduced (16.82%) in worms fed K12 bacteria supplemented with TMA. (Figure 5a). Consistent with our observations in the chemotaxis and dauer formation assays, there was no significant increase in the lifespan of *tyra*‐*3(ok325)* mutant worms fed with *dmsC* mutant bacteria compared to K12‐fed worms (Figure 5b). However, on continuous exposure to TMA (2 mM), *tyra*‐*3(ok325)* mutants had a decreased mean lifespan by ~21% on K12 and 19% on *dmsc* mutant, respectively, implying that there may be additional receptors that mediate the reduction of lifespan by TMA (Figure 5b). No significant increase or decrease in lifespan was observed in *daf*‐*11(m47)* worms fed with *dmsC* bacterial mutant or TMA, respectively (4%), implying an important role for DAF‐11 (Figure 5c). We observed a small increase in lifespan of *daf*‐*16(mu86)* worms fed with *dmsC* bacterial mutant (8.11%). We also observed that TMA treatment reduced the lifespan in *daf*‐*16(mu86)* worms (9.67%) suggesting partial dependence of TMA on DAF‐16 activity in mediating lifespan changes (Figure 5d).

Insulin‐like peptides (ILPs) have been shown to regulate DAF‐16 activity, but are also themselves regulated by DAF‐16 as part of both positive and negative feedback loops (Liu et al., 2004). ILPs also regulate the dauer entry and exit signals by functioning as either agonist or antagonist (Cornils et al., 2011). Certain ILPs are upregulated in *daf*‐*2* mutants raised on *Escherichia coli* OP50 (Mueller et al., 2014). Using qPCR, we tested the expression of ILP genes in N2 worms fed with either K12 or *dmsC* mutant bacteria and found significant upregulation of *ins*‐*35* in *dmsC* fed worms. The increased expression of *ins*‐*35* was rescued by addition of TMA (2 mM) to *dmsC* bacteria (Figure S4a).

We next investigated the role of ILS pathway in dauer formation. Unlike wild‐type N2 worms, *ins*‐*35(ok3297)* mutant worms did not show significant alterations in dauer formation when exposed to TMA in the presence of *dmsC* bacteria compared to worms fed with K12 bacteria (Figure S4b). These results suggest that TMA modulates the expression of insulin‐like peptides, including INS‐35, to influence dauer formation in *C. elegans*. Overall, our results suggest that TMA potentially triggers a neuroendocrine signal that modulates DAF‐16 activity to influence dauer formation and lifespan. These results support the hypothesis that bacteria with reduced TMA levels are sensed in part through TYRA‐3 and DAF‐11 to modulate lifespan in *C. elegans*.

## 3 DISCUSSION

*C. elegans* sense signals from bacteria through ciliated amphid neurons at the tips of their head and phasmid neurons in their tail (Golden & Riddle, 1982, 1984; Jensen et al., 2010; Y. Wang & Levy, 2006). However, the identities of specific bacterial signals that modulate food perception in *C. elegans* remain largely unknown. Here, we demonstrate that *C. elegans* utilizes TMA as one of the signals to detect bacteria's presence. In the HW strain of *C. elegans*, a GPCR, TYRA‐3, regulates lawn‐leaving behavior and is proposed to sense nutrient signals (Bendesky et al., 2011). Our results show that *C. elegans* sense TMA through TYRA‐3, which is expressed in amphid neurons in wild‐type N2 worms. In the HW strain containing a mutated form of *tyra*‐*3*, these animals failed to display ability to sense TMA. *C. elegans* TYRA‐3 has high homology to the human trace amine‐associated receptor 5 (TAAR5) (WormBase). Human TAAR5, a canonical olfactory receptor, has previously been shown to be activated *in vitro* by TMA in heterologous expression systems (Wallrabenstein et al., 2013), supporting a conserved role for these receptors in sensing the bacterial metabolite, TMA. Our data suggest that TYRA‐3 could be a putative GPCR associated with food sensing.

TMA is a colorless tertiary amine and has a strong “fishy” odor (Messenger et al., 2013). In humans, TMA is produced by gut bacterial metabolism and is associated with trimethylaminuria (odorous sweat), bad breath vaginal odor due to bacterial vaginosis, and pathology of AD (Messenger et al., 2013). Bacterial TMAO and DMSO reductases are both shown to reduce TMAO to TMA, but they are genetically distinct from each other. *E. coli* DMSO reductase is a trimeric enzyme composed of an integral membrane anchor (*dmsC*) and a catalytic dimer (*dmsAB*). These enzymes catalyze the reduction of a number of S‐ and N‐oxide compounds, including DMSO and TMAO. DMSO reductase activity is conserved among a wide variety of micro‐organisms including prokaryotes and eukaryotes, aerobes and anaerobes (Zinder & Brock, 1978). Our results demonstrate that single‐gene mutant bacteria strains from the Keio library (Baba et al., 2006) can be used in conjunction with *C. elegans* to successfully identify physiologically relevant bacterial signals, including different type of amines. We demonstrated here that TMA from bacteria acts as a nutrient signal for *C. elegans* that influences development by activation of a conserved guanylyl cyclase pathway in neurons and regulates the ILS pathway through the peptide INS‐35. TMA‐derived metabolites are associated with increased risk for type II diabetes, cardiovascular disease, and atherosclerosis in humans (Gipson et al., 2008; Z. Wang et al., 2011). Inhibiting the production of TMA by targeting the microbial enzymes is considered as potential therapeutic approach for TMA‐associated metabolic diseases (Z. Wang et al., 2015). Given the importance of nutrient signaling pathways in modulating these diseases, we speculate that TMA may also modulate these conserved signaling pathways to alter host physiology in higher organisms.

**FIGURE 6:**
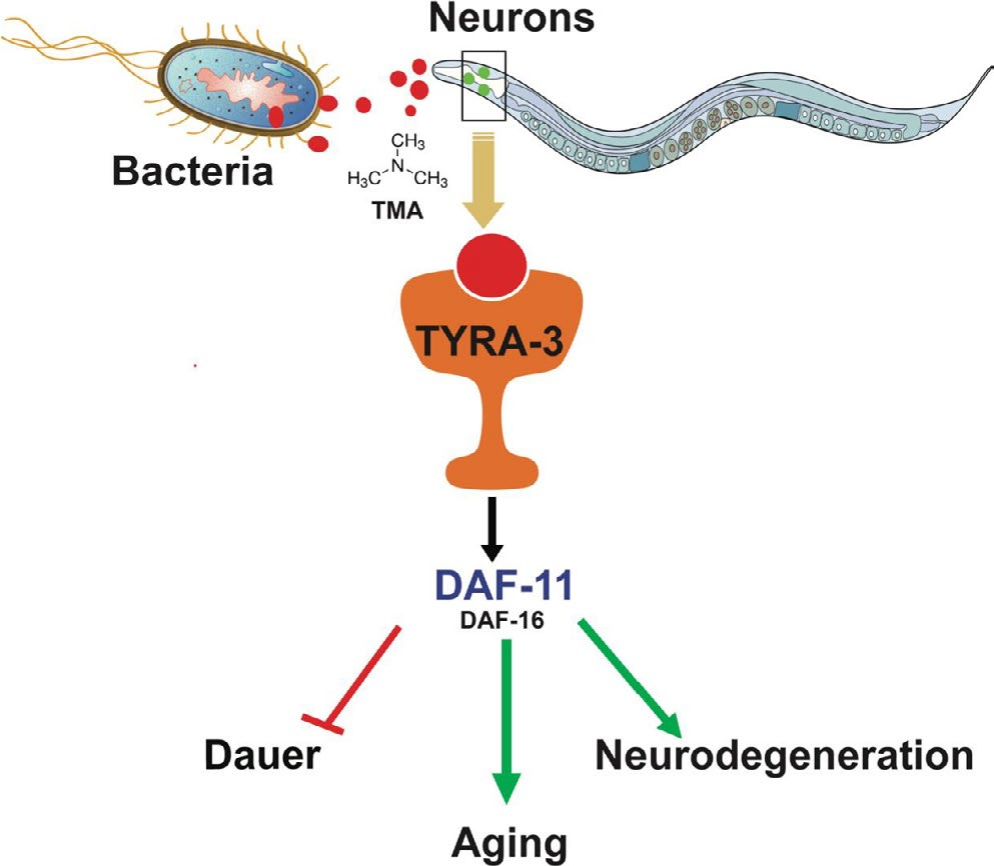
TMA alters development, aging, and neurodegeneration in *C. elegans*. Observed results suggests that the bacterial metabolite TMA alters development, aging, and neurodegeneration in *C. elegans* through TYRA‐3, DAF‐11 and DAF‐16 pathways

Microbes play a critical role in various host functions, including nutrient responses and immunity, and have also been linked to various human diseases (J. Wang et al., 2013). Many species have evolved to have a commensal relationship with microbes by physically harboring certain microbial species, while some species like *C. elegans* possess elaborate systems to seek these microbial products from their environment as a source of nutrients. Our data suggest that bacterial signals can directly modulate nutrient signaling pathways critical for development, aging, and neurodegeneration in worms (Figure 6), pathways which are also strongly linked with diseases such as atherosclerosis and diabetes in humans. The evolution of the gut from a relatively simple tube in ancient cyclostomatida to the complex gastrointestinal tract of mammalian species has led to a concomitant increase in diversity and complexity of microbial populations. The use of *C. elegans* with a reduced microbial complexity can help understand how specific bacterial metabolites can regulate host physiology and behavior.

## 4 MATERIALS AND METHODS

### 4.1 *C. elegans* strains and growth conditions

All strains were obtained from the *Caenorhabditis* Genetics Center (CGC). The strains obtained from the CGC were outcrossed 6 times to N2 (wild‐type) worms. Strains used in this manuscript from CGC are as follows: *daf‐2(e1370), daf‐7(e1372), daf‐11(m47), daf‐16(mu86), DAF‐16::GFP (daf‐16(mgDf47)), ser‐7(tm1325), ser‐5(ok3087), tyra‐3(ok325), odr‐4(n2144)*. Cori Bargmann kindly provided us with N2‐*tyra*‐*3* rescue strains (Bendesky et al., 2011) CX13112‐CX13114 *kyEx3778*‐*kyEx3780* [P*sri*‐*51*::N2‐*tyra*‐*3b*::SL2 GFP], CX11495‐CX11497 *kyEx3063*‐*kyEx3065* [P*sra*‐*9*::N2‐*tyra*‐*3b*::SL2 GFP], CX13118‐CX13120 *kyEx3784*‐*kyEx3786* [P*flp*‐*17*::N2‐*tyra*‐*3b*::SL2 GFP], CX13115‐CX13117 *kyEx3781*‐*kyEx3783* [P*dat*‐*1*::N2‐*tyra*‐*3b*::SL2 GFP], and *tax*‐*4(p678)*;*daf*‐*11(m47)*. Strain UA44 [baInl1 (P*_dat_*_‐_ *_1_*::αsyn, P*_dat_*_‐_ *_1_*::GFP)] was kind gift from Guy Caldwell, University of Alabama. UA44 is overexpresser of α‐synuclein, and it is denoted as αsyn OE in this manuscript. Strain αsyn OE;*tyra*‐*3* is UA44 strain with *tyra*‐*3* mutation was generated by conventional crossing protocol that resulted in strain genotype [baInl1 (P*_dat_*_‐_ *_1_*::αsyn, P*_dat_*_‐_ *_1_*::GFP); *tyra*‐*3(ok325)*]. Strains were maintained on nematode growth media plates at 20°C seeded with *E. coli* *(K12)* bacteria grown in Luria broth.

### 4.2 *E. coli* strains

*E. coli* mutant strains, including the parent K12 BW25113 strain, were obtained from the Keio mutant collection (Baba et al., 2006). The strain library was shipped and stored as a frozen stock in 96‐well plates. Stock plates were pin‐replicated to produce a working copy plate containing Luria Broth (LB) and 25 μg/ml kanamycin. For assays used in this study, *E. coli* strains were stab‐cultured and grown overnight with shaking at 37°C in minimal media as described below. Overnight cultures were seeded on minimal media plates and dried at 37°C for 12 hours prior to transfer of *C. elegans*.

### 4.3 *C. elegans* and *E. coli* media

Luria broth (LB) and nematode growth media (NGM) were used to maintain stock cultures of *E. coli* and *C. elegans* strains, respectively. Minimal media was used for assays in this study as described previously (Kim et al., 2014). Liquid media for bacterial growth contained 0.05 M NaCl, 0.04 M NH_4_Cl, 0.01 M CaCl_2_, 0.025 M phosphate buffer, 0.4% glucose, 1 µg/mL thiamine, and 0.001 M MgSO_4_. Minimal media plates for subsequent *C. elegans* experiments contained 2% agar, 0.05 M NaCl, 0.04 M NH_4_Cl, 0.001 M CaCl_2_, 1 µg/mL cholesterol, 0.025 M phosphate buffer, 0.4% glucose, 1 µg/mL thiamine, and 0.001 M MgSO_4._ Trimethylamine (TMA) for experiments was purchased from Sigma‐Aldrich: Trimethylamine solution (~45 wt. % in H_2_O).

### 4.4 PCR confirmation of *dmsC* mutant

Bacterial strains for kanamycin cassette confirmation were streaked onto LB plates containing 25 μg/ml kanamycin. Strains were colony‐purified, and genomic PCR was performed as follows: Primer sets were developed for individual knockout strains encompassing sequences from outside the target gene. The forward primer was designed to be within 200 bp upstream of the 5’ end and the reverse primer to be within 200 bp downstream of the 3’ end of the target gene. Thermal cycler parameters were as follows: initial 5 min at 94°C, then 25 cycles of 30 s at 94°C, 10 s at 56°C, 1 min at 72°C, and final 5 min at 72°C. Products were run on an agarose gel, and amplified products were examined for appropriate length.

**Table:** 

*dmsC*	Forward Primer	ATCGCCTTCTATCGCCTTCTTAGC
	Reverse Primer	CGTCTTGATAAAAATGATGCTGCC

### 4.5 RNA isolation and qPCR validation

Quantitative real‐time PCR was carried out using SYBR Green I (Sigma‐Aldrich) assay reagents to verify gene expression profiles. Two hundred nanograms of total RNA were reverse transcribed to cDNA; real‐time PCR reactions were performed on a 7500 Fast System Real‐Time PCR cycler (Applied Biosystems, Foster City, CA), according to the manufacturer's instructions. Changes in gene expression between different treatment groups were calculated using the delta Ct method.

Primer sequences:

**Table:** 

*tyra−3*	Forward Primer	AGCACGACTGCCACAATTA
	Reverse Primer	GTGGAGAGTTCGAGCCTAATG
*ins−35*	Forward Primer	ACGCGCACTAAAGGTCTATTC
	Reverse Primer	GTTGAGCCACATCCTTCCAT
*act−1*	Forward Primer	CAACACTGTTCTTTCCGGAG
	Reverse Primer	CTTGATCTTCATGGTTGATGGG

### 4.6 Dauer assay

*Dauer assay using “no*‐*glucose” conditions*: To determine whether TMA reduces dauer formation, dauer assays were performed in non‐permissive temperature conditions (27°C for N2 wild‐type and 21.5ºC for the other Daf‐c mutants) with no glucose added to the minimal media plates with *E. coli* (K12) bacteria overnight culture seeded on the plates. TMA was added on top of the bacterial lawn and spread around the entire breadth of the plate at a final concentration of 10 mM and air‐dried for 15 minutes at 20°C without lids. The eggs were placed on the bacterial lawn and incubated at respective temperatures for 4–5 days prior to assessing numbers of dauer versus non‐dauer state worms. Dauers were selected by treatment with 1% sodium dodecyl sulfate (SDS) for 15 minutes, transferred to a fresh plate, and counted.

*Dauer assay using bacterial mutants*: Bacterial strains were grown to stationary phase in minimal medium prior to being seeded on minimal media agar. Mutant and control *E. coli* plates were left to grow overnight at 37ºC. Eggs were placed on plates containing *E. coli* mutants. Plates were incubated at 21.5ºC for 4–5 days prior to quantitating dauer versus non‐dauer state worms. Worm larval states were analyzed using a dissecting microscope and manually counted.

### 4.7 Chemotaxis assay

Each chemotaxis assay plate contained 1 mM MgSO4, 1 mM CaCl2, 5 mM potassium phosphate pH 6.0, and 2% agar in a 6 cm petri dish. Plates were air‐dried and stored overnight at 4°C. Prior to the assay, 4 µl of 2 mM TMA and control solvent (water) was freshly spotted equidistant from the center of the plate and was air‐dried at 20°C for 10 minutes without lids. Age‐synchronized day 1 adult worms were washed and prepared as previously described (Glater et al., 2014). Worms were placed in the center of the plate, and positions of the worms were observed using stereomicroscope and images taken at 15‐minute intervals. The chemo‐attraction index was documented as described previously (Lans et al., 2004).

### 4.8 Bacterial choice assay

Age‐synchronized day 1 adult worms and minimal media plates were used for this assay. Plates include 2% agar, 0.05 M NaCl, 0.04 M NH_4_Cl, 0.001 M CaCl_2_, 5 µg/mL cholesterol, 0.025 M phosphate buffer, 0.4% glucose, 1 µg/mL thiamine, and 0.001 M MgSO_4._ Overnight bacterial culture with OD 600 nm of 2.0 was spotted (30 µl) equidistant from the center of the plate and dried for 15 minutes at 37°C. Both K12 and *dmsC* mutant bacteria had approximately the same cellular density: K12 bacteria at 0.8 × 10^7^ ± 1.2 × 10^7^ colony‐forming units (cfu) per ml and *dmsC* mutant bacteria at 0.7 × 10^7^ ± 1.3 × 10^7^ colony‐forming units (cfu) per ml. Worms were washed and prepared as previously described (Glater et al., 2014). The worms were placed in the center of the plate, and positions of the worms were observed using stereomicroscope and images taken at 15‐minute interval. The bacterial choice index was documented as described before (Glater et al., 2014).

### 4.9 Lawn‐leaving assay

Age‐synchronized day 1 adult animals (*n* = 20 per plate) were transferred to bacterial lawns cultured under conditions as described above. Assay was performed for 5 minutes, and lawn‐leaving events were manually counted by observing under the microscope and averaged to lawn‐leaving events per minute as previously described (Bendesky et al., 2011).

### 4.10 Fluorescence microscopy

Age‐synchronized L1‐stage animals carrying DAF‐16::GFP integrated transgenes (*daf*‐*16(mgDf47)*) were placed on plates seeded with overnight cultures of bacterial strains as described above. Animals were grown at 15°C for 2–3 days and changed to higher temperature for one day, unless otherwise noted. The fluorescence intensity and/or location was captured using reflected light fluorescence microscopy (Olympus IX3), and the images were processed for densitometry analysis using ImageJ.

### 4.11 Lifespan assay

Late L4 larvae growing at 15°C were transferred to fresh minimal media plates with FUdR (5 µg/ml) added to the bacterial lawn as indicated in “Results.” The first day of adulthood is designated as day 1 in survival curves. Animals were scored as alive, dead, or lost every other day. Animals that failed to display touch‐provoked movement were scored as dead. Animals that died from causes other than aging, such as sticking to the plate walls, internal hatching, or bursting in the vulval region, were scored as lost. Animals were transferred to fresh plates and fresh *E. coli* every 2 days with TMA added to have final concentration 10 mM. Plates were dried for 15 minutes at 20°C before the worms were transferred. All lifespan experiments were performed at 21.5°C. Survival curves were plotted, and statistical analyses (log‐rank tests) were performed using Prism 6 software (GraphPad Software, Inc., San Diego, CA, USA). Statistical significance between lifespan curves was determined by p‐value of <0.05.

### 4.12 RNAi knockdown of gene expression

RNAi bacterial strains expressing double‐stranded RNA that inactivates specified genes were cultured and used as previously described (Timmons et al., 2001). Briefly, eggs isolated from synchronous populations of cultures were placed on fresh RNAi IPTG and antibiotic‐supplemented plates and allowed to grow at 15°C; 3 days later, L4 molt nematodes were used for assays as mentioned in “Results.”

### 4.13 Pharyngeal Pumping

Worms were observed using stereomicroscope at 40x magnification. Pumps per minute were counted as described previously (Raizen et al., 2012).

### 4.14 Statistical Analysis

Student's two‐tailed *t test* was used when comparing two sets of data. One‐way ANOVA and multiple comparison test were performed for comparing between groups using Prism 6.0. Survival curves were generated using Prism 6.0, and statistical analysis was performed using log‐rank t test.

## CONFLICT OF INTEREST

The authors declare no conflict.

## AUTHOR CONTRIBUTIONS

AK and PK designed the study. AK, MV, and JK did the dauer and lifespan assays. MC helped with transgenic strain generation. DS analyzed data, drafted manuscript, and addressed reviewers’ queries. SC and AR helped with data analysis. CN and RB helped with expression data analysis. HP, JA, and GL gave valuable inputs in the drafting of the manuscript.

## Supporting information

Figure S1‐S4

Table S1‐S3

## Data Availability

The data that supports the findings of this study are available in the supplementary material of this article.
