# Supplementary material for: Trimethylamine modulates dauer formation, neurodegeneration, and lifespan through tyra‐3/daf‐11 signaling in Caenorhabditis elegans
Source: Aging Cell. 2021 Apr 5;20(5):e13351. doi: 10.1111/acel.13351 (PMC8135002; doi:10.1111/acel.13351)
Supplement: Supplementary file 1 — Figure S1‐S4 [file ACEL-20-e13351-s002.pdf]

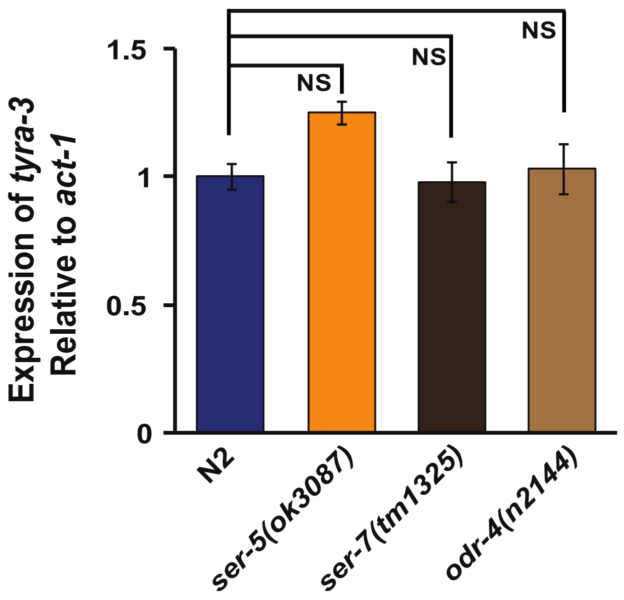

Figure S1. Worms does not require *ser-5 ser-7 or odr-4* for sensing TMA A. Gene expression of *tyra-3* was determined in age-synchronized L1 stage *worms feed with tyra-2*(RNAi) bacteria. Expression of *tyra-3* was also quantitated in *ser-5(ok3087)*, *ser-7(tm1325)* and *odr-4(n2144)* mutant worms.

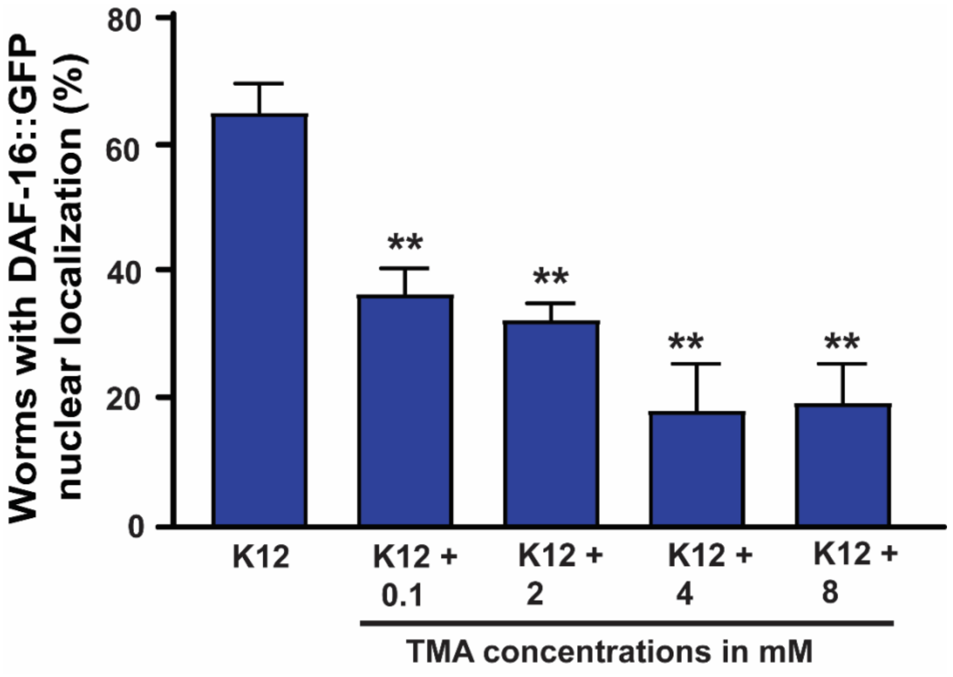

Figure S2. TMA reduces nuclear localization of DAF-16::GFP. In the presence of TMA, retention of DAF-16 in the cytoplasm was observed in DAF-16::GFP worms. Histogram showing the percent of worms positive for nuclear localization; samples were treated with 0.1 to 8 mM TMA. Worms n~300, n≥ 6 assays. **p<0.01; average ± std. dev (n=3).

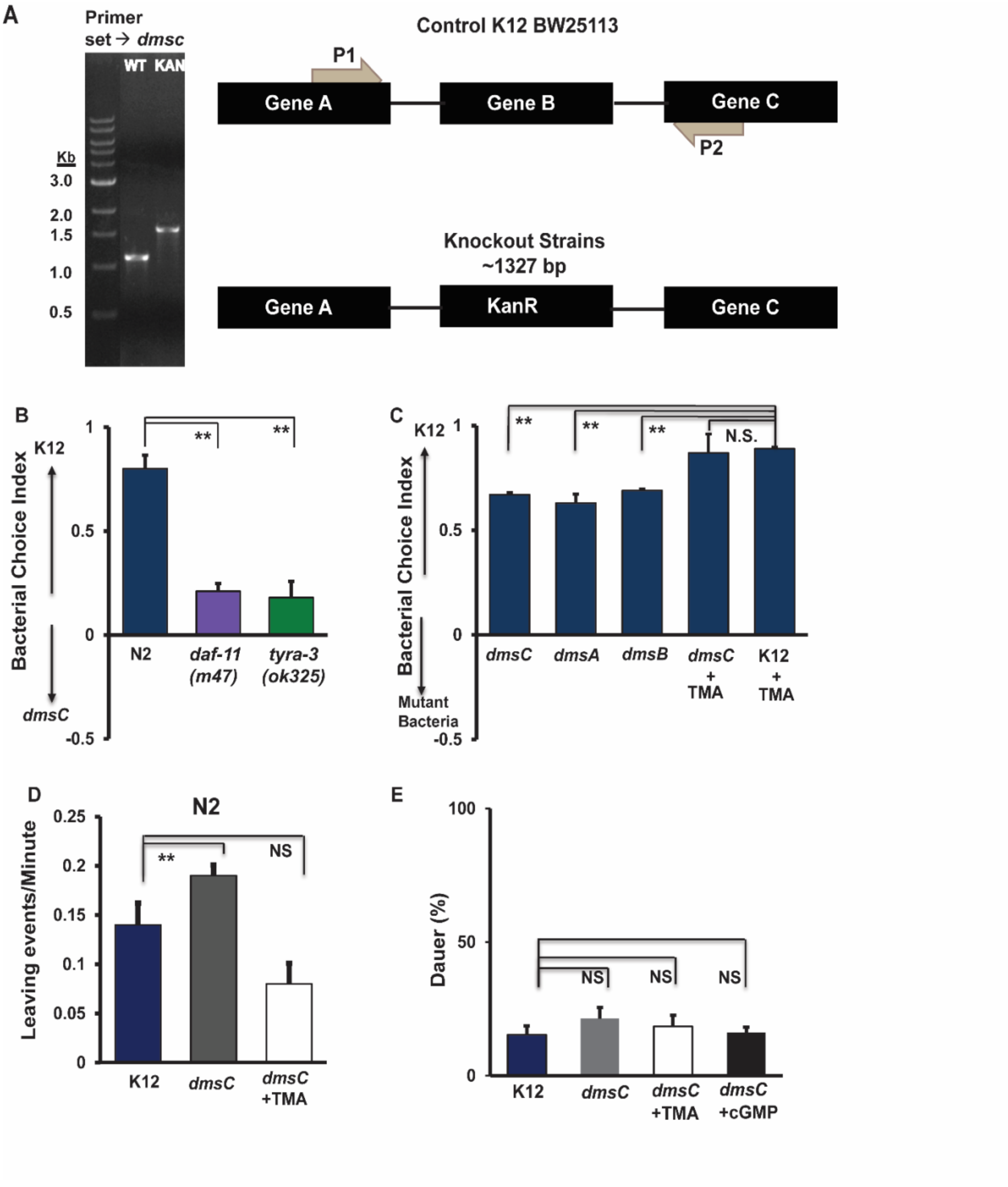

Figure S3. Bacteria-specific DMSO reductase mutant modulates nutrient sensing and dauer formation in *C. elegans:* A. Confirmation of *dmsC* knockout in Keio library using PCR assay and *dms* mutation details. **B.** N2 worms showed positive chemo-attraction towards K12 lawn as compared to *dmsC* mutant bacterial lawn. *daf-11(m47)* and *tyra-3(ok325)* mutants did not show any preference for K12 bacterial lawn. Bacterial choice index was calculated after 30 minutes of transferring the animals to assay plates.

C. Worms showed a preference towards K12 bacteria *vs dmsC* mutant bacteria. **D.** An increase in lawn-leaving events was observed in worms fed *dmsC* mutant bacterial lawns and a significant rescue of this phenotype was observed in worms fed on *dmsC* mutant bacterial lawns in the presence of 2 mM TMA. **E.** Dauer assays were performed on minimal media plates without glucose on K12 bacterium lawns at 27°C in the presence and absence of TMA (2 mM) and cGMP (2 mM). Synchronized egg population from *daf-11(m47),* and *tax-4;daf-11* double mutant was used. Worms n~300, n≥ 6 assays. **p<0.01; average ± std. dev (n=3).

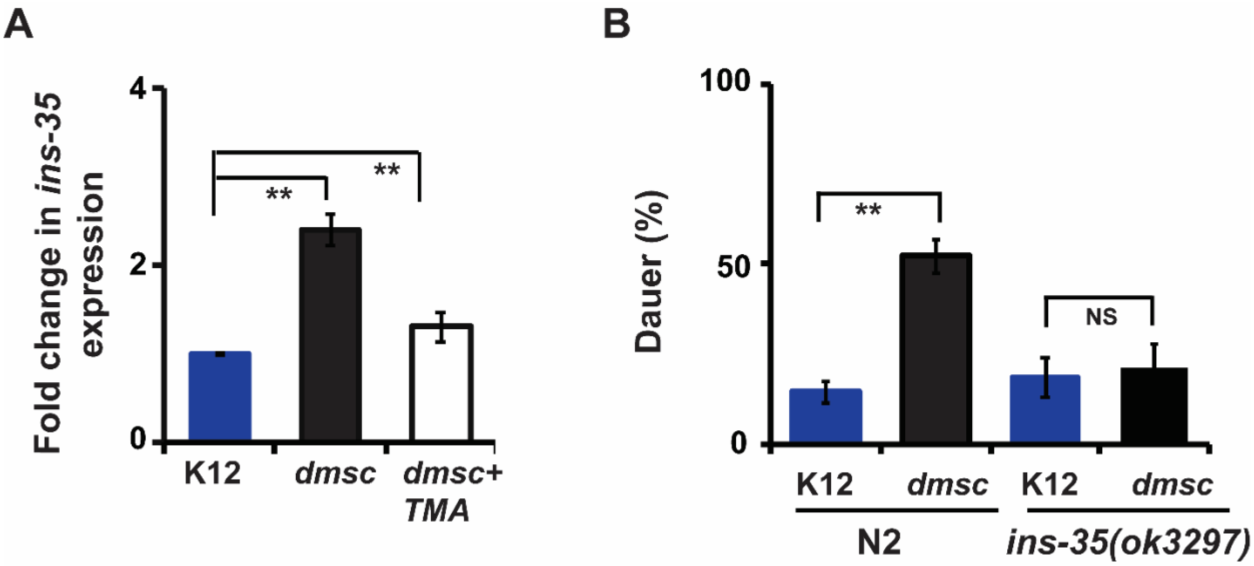

Figure S4: TMA modulates dauer formation by altering expression of insulin like peptides (ILPs): A. Expression of *ins-35* gene in age-synchronized late L1 (N2) worms fed K12 in the presence or absence of TMA (2 mM). **B.** Dauer assays were performed on minimal media plates without glucose fed K12 in the presence or absence of TMA (2 mM). Synchronized egg population from *ins-35(ok3297)* mutant worms was used. **p<0.01; average ± std. dev (n=3).
