# Supplementary material for: Trimethylamine modulates dauer formation, neurodegeneration, and lifespan through tyra‐3/daf‐11 signaling in Caenorhabditis elegans
Source: Aging Cell. 2021 Apr 5;20(5):e13351. doi: 10.1111/acel.13351 (PMC8135002; doi:10.1111/acel.13351)
Supplement: Supplementary file 2 — Table S1‐S3 [file ACEL-20-e13351-s001.docx]

**Supplementary Information:**

# Trimethylamine modulates dauer formation, neurodegeneration, and lifespan through tyra‐3/daf‐11 signaling in Caenorhabditis elegans

| **Table S1a: Statistical analysis of chemoattraction and bacterial choice experiments** | | | | | |
| --- | --- | --- | --- | --- | --- |
|  |  |  |  |  |  |
| **Genotype** | **Bacteria ^a^** | **Chemical** | **CI** | **n ^b^** | ***p* ^c^** |
|  |  |  | **Average** |  |  |
| **Worms show positive chemoattraction toward TMA.** | | | |  |  |
| N2 | / | TMA | 0.07 | 300 | -0.020 |
| N2 | / | TMA 0.05mM | 0.32 | 300 |  |
| N2 | / | TMA 0.5mM | 0.49 | 300 | 0.022 |
| N2 | / | TMA 1mM | 0.58 | 300 | 0.011 |
| N2 | / | TMA 5mM | 0.64 | 300 | 0.004 |
| N2 | / | TMA 10mM | 0.80 | 300 | 0.000 |
| N2 | / | TMA 20mM | 0.78 | 300 | 0.000 |
|  |  |  |  |  |  |
| N2 | / | TMA 2mM | 0.89 | 300 | 0.001 |
| *daf-11(m47)* | / | TMA 2mM | -0.06 | 300 |  |
| *odr-4(n2144)* | / | TMA 2mM | 0.87 | 300 | 0.000 |
| *tyra-3(ok325)* | / | TMA 2mM | 0.14 | 300 | 0.122 |
| *tyra-2(RNAi)* | / | TMA 2mM | 0.22 | 300 | 0.083 |
| *ser-5(ok3087)* | / | TMA 2mM | 0.81 | 300 | 0.000 |
| *ser-7(tm1325)* | / | TMA 2mM | 0.81 | 300 | 0.000 |
|  |  |  |  |  |  |
| N2 | / | TMA 2mM | 0.74 | 300 | 0.000 |
| HW | / | TMA 2mM | 0.18 | 300 |  |
| ASK | / | TMA 2mM | 0.65 | 300 | 0.001 |
| BAG | / | TMA 2mM | 0.53 | 300 | 0.006 |
| CEP | / | TMA 2mM | 0.21 | 300 | 0.592 |
| ADL | / | TMA 2mM | 0.12 | 300 | 0.102 |
| HW-N2TYRA-3 | / | TMA 2mM | 0.64 | 300 | 0.001 |
|  |  |  |  |  |  |
| **Table S1b: Worms show preference towards K12 bacteria *vs* *dmsC* mutant bacteria** | | | | | |
| **Genotype** | **Bacteria^a^** | **Chemical** | **CI** | **n ^b^** | ***p* ^c^** |
|  |  |  |  |  |  |
|  |  |  | **Average** |  |  |
| N2 | K12/*dmsC* | / | 0.56 | 300 | 0.004 |
| HW | K12/*dmsC* | / | 0.02 | 300 |  |
| ASK | K12/*dmsC* | / | 0.40 | 300 | 0.004 |
| BAG | K12/*dmsC* | / | 0.33 | 300 | 0.013 |
| CEP | K12/*dmsC* | / | 0.35 | 300 | 0.004 |
| ADL | K12/*dmsC* | / | 0.03 | 300 | 0.788 |
| HW-N2TYRA-3 | K12/*dmsC* | / | 0.35 | 300 | 0.003 |

**Table S2a:** **Statistical analysis of dauer assay experiments**

| **Genotype** | **Bacteria ^a^** | **Chemical** | **Dauer** | **n ^b^** | ***p* ^c^** |
| --- | --- | --- | --- | --- | --- |
|  |  |  | **Average** |  |  |
|  |  |  |  |  |  |
| **TMA inhibits dauer formation through *daf-11/*guanyl cyclase *signaling* pathway** | | | | | |
| N2 | K12 | / | 66.54 | 218 | 0.000 |
| N2 | K12 | TMA | 43.89 | 226 |  |
| *daf-2(e1370)* | K12 | / | 83.92 | 236 | 0.000 |
| *daf-2(e1370)* | K12 | TMA | 43.69 | 206 |  |
| *daf-7(e1368)* | K12 | / | 54.74 | 201 | 0.001 |
| *daf-7(e1368)* | K12 | TMA | 36.90 | 192 |  |
| *daf-11(m47)* | K12 | / | 49.81 | 227 | 0.217 |
| *daf-11(m47)* | K12 | TMA | 47.23 | 226 | 0.004 |
| *daf-11(m47)* | K12 | TMA, cGMP | 26.15 | 197 |  |
| *tax-4(p678);daf-11(m47)* | K12 | / | 50.64 | 212 | 0.377 |
| *tax-4(p678);daf-11(m47)* | K12 | TMA | 48.41 | 225 | 0.392 |
| *tax-4(p678);daf-11(m47)* | K12 | TMA, cGMP | 50.96 | 216 |  |
| N2 | K12 | / | 65.14 | 224 | 0.910 |
| N2 | K12 | TMA, 0.1mM | 65.53 | 227 |  |
| N2 | K12 | TMA, 0.2mM | 54.09 | 256 |  |
| N2 | K12 | TMA, 0.4mM | 43.74 | 220 | 0.000 |
| N2 | K12 | TMA, 0.8mM | 53.35 | 218 |  |
| N2 | K12 | / | 68.50 | 242 | 0.001 |
| N2 | K12 | TMAO, 0.5mM | 24.50 | 165 |  |
|  |  |  |  |  |  |
| **Table S2b: Disruption of bacterial enzyme involved in TMA production alters dauer formation** | | | | | |
| N2 | K12 | / | 8.07 | 234 | 0.000 |
| N2 | *dmsC* | / | 53.34 | 214 |  |
| *daf-2(e1370)* | K12 | / | 13.37 | 225 | 0.000 |
| *daf-2(e1370)* | *dmsC* | / | 56.80 | 213 |  |
| *daf-7(e1368)* | K12 | / | 17.29 | 226 | 0.000 |
| *daf-7(e1368)* | *dmsC* | / | 51.70 | 236 |  |
| *daf-11(m47)* | K12 | / | 14.48 | 226 | 0.880 |
| *daf-11(m47)* | *dmsC* | / | 14.86 | 215 |  |
| N2 | K12 | / | 8.02 | 235 | 0.000 |
| N2 | *dmsC* | / | 55.21 | 214 |  |
| N2 | K12 | TMA | 7.61 | 222 | 0.026 |
| N2 | *dmsC* | TMA | 23.17 | 227 |  |
| *tyra-3(ok325)* | K12 | / | 13.15 | 228 | 0.062 |
| *tyra-3(ok325)* | *dmsC* | / | 17.57 | 228 |  |
| *tyra-3(ok325)* | K12 | TMA | 16.42 | 225 | 0.591 |
| *tyra-3(ok325)* | *dmsC* | TMA | 14.98 | 238 |  |
| N2 | K12 | / | 11.77 | 238 | 0.000 |
| N2 | *dmsA* | / | 46.14 | 203 |  |
| N2 | *dmsA* | TMA | 21.12 | 218 | 0.001 |
| N2 | *dmsB* | / | 44.06 | 211 |  |
| N2 | *dmsB* | TMA | 22.72 | 207 |  |
| N2 | *betA* | / | 26.38 | 235 | 0.016 |
| N2 | *betA* | TMA | 17.45 | 226 |  |
| N2 | *betB* | / | 19.98 | 234 | 0.104 |
| N2 | *betB* | TMA | 14.09 | 233 |  |
| N2 | *torA* | / | 46.17 | 243 | 0.000 |
| N2 | *torA* | TMA | 20.29 | 215 |  |
| N2 | *torC* | / | 47.81 | 236 | 0.006 |
| N2 | *torC* | TMA | 25.19 | 228 |  |
| N2 | *torY* | / | 47.48 | 230 | 0.002 |
| N2 | *torY* | TMA | 22.62 | 216 |  |
| N2 | *torZ* | / | 46.84 | 221 | 0.000 |
| N2 | *torZ* | TMA | 23.73 | 236 |  |
| N2 | K12 | / | 10.07 | 237 | 0.000 |
| N2 | *dmsC* | / | 54.25 | 220 |  |
| N2 | K12 | Choline, 0.5mM | 13.03 | 224 | 0.177 |
| N2 | *dmsC* | Choline, 0.5mM | 30.18 | 242 | 0.004 |
| N2 | K12 | TMAO, 0.5mM | 12.70 | 244 | 0.052 |
| N2 | *dmsC* | TMAO, 0.5mM | 21.38 | 239 | 0.007 |
| *tyra-3(ok325)* | K12 | / | 15.27 | 229 | 0.477 |
| *tyra-3(ok325)* | *dmsC* | / | 17.91 | 245 |  |
| *tyra-3(ok325)* | K12 | Choline, 0.5mM | 13.14 | 209 | 0.670 |
| *tyra-3(ok325)* | *dmsC* | Choline, 0.5mM | 13.99 | 242 | 0.326 |
| *tyra-3(ok325)* | K12 | TMAO, 0.5mM | 15.07 | 228 | 0.905 |
| *tyra-3(ok325)* | *dmsC* | TMAO, 0.5mM | 12.91 | 202 | 0.975 |
|  |  |  |  |  |  |
| **Table S2c: Dauer Assay: Transgenic HW strain with TYRA-3 neuronal expression** | | | | | |
| N2 | K12 | / | 14.70 | 224 | 0.000 |
| N2 | *dmsC* | / | 53.77 | 214 |  |
| N2 | K12 | TMA | 13.83 | 188 | 0.648 |
| N2 | *dmsC* | TMA | 13.39 | 187 |  |
| HW | K12 | / | 14.98 | 207 | 0.127 |
| HW | *dmsC* | / | 19.25 | 214 |  |
| HW | K12 | TMA | 13.10 | 185 | 0.396 |
| HW | *dmsC* | TMA | 14.61 | 164 |  |
| HW-N2*tyra-3*-ASK | K12 | / | 14.12 | 182 | 0.000 |
| HW-N2*tyra-3*-ASK | *dmsC* | / | 39.33 | 224 |  |
| HW-N2*tyra-3*-ASK | K12 | TMA | 13.95 | 142 | 0.236 |
| HW-N2*tyra-3*-ASK | *dmsC* | TMA | 16.87 | 154 |  |
| HW-N2*tyra-3*-BAG | K12 | / | 15.26 | 210 | 0.000 |
| HW-N2*tyra-3*-BAG | *dmsC* | / | 35.11 | 188 |  |
| HW-N2*tyra-3*-BAG | K12 | TMA | 17.68 | 137 | 0.732 |
| HW-N2*tyra-3*-BAG | *dmsC* | TMA | 17.01 | 147 |  |
| HW-N2*tyra-3*-CEP | K12 | / | 14.52 | 179 | 0.000 |
| HW-N2*tyra-3*-CEP | *dmsC* | / | 28.35 | 194 |  |
| HW-N2*tyra-3*-CEP | K12 | TMA | 17.70 | 192 | 0.000 |
| HW-N2*tyra-3*-CEP | *dmsC* | TMA | 12.96 | 201 |  |
| HW-N2*tyra-3*-ADL | K12 | / | 17.57 | 193 | 0.662 |
| HW-N2*tyra-3*-ADL | *dmsC* | / | 18.53 | 194 |  |
| HW-N2*tyra-3*-ADL | K12 | TMA | 19.45 | 180 | 0.288 |
| HW-N2*tyra-3*-ADL | *dmsC* | TMA | 18.13 | 182 |  |

Dauer assays were performed under experimental conditions described in the methods section. a, bacterial strain; b, numbers of animals scored; c, log-rank tests

**Table S3: Lifespan assay**

| **Genotype** | **Bacteria ^a^** | **Chemical** | **Lifespan (days)** | | **Percent Change ^b^** | **n ^c^** | ***p* ^d^** |
| --- | --- | --- | --- | --- | --- | --- | --- |
|  |  |  | **Mean** | **Max** |  |  |  |
| N2 | K12 | / | 14.20 | 20 | / | 102 | / |
| N2 | *dmsC* | / | 16.41 | 24 | 15.61 | 102 | < 0.0001 |
| N2 | K12 | TMA | 11.81 | 20 | -16.82 | 74 | 0.0002 |
| N2 | *dmsC* | TMA | 13.79 | 22 | -2.89 | 78 | < 0.0001 |
| *tyra-3(ok325)* | K12 | / | 14.14 | 22 | / | 91 | / |
| *tyra-3(ok325)* | *dmsC* | / | 14.71 | 24 | 0.00 | 93 | 0.2973 |
| *tyra-3(ok325)* | K12 | TMA | 11.50 | 18 | -21.84 | 90 | < 0.0001 |
| *tyra-3(ok325)* | *dmsC* | TMA | 11.85 | 18 | -19.44 | 92 | < 0.0001 |
| *daf-11(m47)* | K12 | / | 28.51 | 36 | / | 93 | / |
| *daf-11(m47)* | *dmsC* | / | 27.26 | 36 | -4.38 | 91 | 0.6314 |
| *daf-11(m47)* | K12 | TMA | 27.09 | 36 | -4.98 | 94 | 0.1931 |
| *daf-11(m47)* | *dmsC* | TMA | 27.23 | 36 | -4.51 | 92 | 0.0257 |
| *daf-16(mu86)* | K12 | / | 12.97 | 18 | / | 97 | / |
| *daf-16(mu86)* | *dmsC* | / | 14.02 | 24 | 8.11 | 99 | 0.0027 |
| *daf-16(mu86)* | K12 | TMA | 11.71 | 20 | -9.67 | 98 | 0.0009 |
| *daf-16(mu86)* | *dmsC* | TMA | 11.62 | 20 | -10.44 | 99 | < 0.0001 |
| Lifespan assays were performed under experimental conditions described in the methods section. a, bacterial strain; b, percentage change in lifespan; c, numbers of animals scored; d, log-rank tests. | | | | | | | |
